# Troxerutin alleviates hepatorenal toxicity induced by carbon tetrachloride in male mice

**DOI:** 10.22038/ijbms.2025.84752.18337

**Published:** 2025

**Authors:** Elham Hakimizadeh, Ayat Kaeidi, Iman Fatemi, Ali Shamsizadeh, Jalal Hassanshahi

**Affiliations:** 1 Physiology-Pharmacology Research Center, Research Institute of Basic Medical Sciences, Rafsanjan University of Medical Sciences, Rafsanjan, Iran; 2 Department of Physiology and Pharmacology, School of Medicine, Rafsanjan University of Medical Sciences, Rafsanjan, Iran; 3 Research Center of Tropical and Infectious Diseases, Kerman University of Medical Sciences, Kerman, Iran

**Keywords:** Apoptosis, Carbon tetrachloride, Mice, Oxidative stress, Troxerutin

## Abstract

**Objective(s):**

Troxerutin (TRX) is a natural bioflavonoid with several medicinal properties. We assessed its protective effect on carbon tetrachloride-related hepatorenal damage in male mice.

**Materials and Methods:**

Male mice were assigned to five groups: Control, TRX, CCL4, CCL_4_ + TRX 75 mg/kg, CCL_4_ + TRX 150 mg/kg. Animals received oral TRX (75 and 150 mg/kg) daily for four weeks. After treatments, serum liver enzymes aspartate aminotransferase (AST), Alanine aminotransferase (ALT), Serum blood urine nitrogen (BUN), and creatinine (Cr) levels were assessed. Malondialdehyde (MDA, the primary lipid peroxidation product), activity of anti-oxidant enzymes glutathione peroxidase (GPX), superoxide dismutase (SOD), and total anti-oxidant capacity (TAC) were determined. Using the immunoblotting method, Bax, Bcl-2, cleaved caspase-3, and cytochrome-c protein concentrations were evaluated in the kidney and liver tissues. The Hematoxylin and Eosin (H&E) staining were used to assess the kidney and liver histopathological changes.

**Results:**

CCl_4 _caused a significant increase in the concentrations of liver and kidney indices such as ALT (*P*<0.05), Cr (*P*<0.01), AST (*P*<0.001), and BUN (*P*<0.001). Furthermore, CCl_4_ significantly increased the MDA level in the liver (*P*<0.01) and kidney (*P*<0.001) tissues while decreasing anti-oxidant status. TRX could significantly decrease ALT, AST, Cr, BUN, and MDA concentrations and increase SOD, GPx, and TAC activities in comparison to the CCl_4_-damaged control group. In addition, TRX caused an attenuation in the pro and anti-apoptotic markers in the kidney and liver tissues.

**Conclusion:**

TRX displayed liver and kidney protection, possibly by its free radical scavenging and anti-oxidant effects.

## Introduction

Carbon tetrachloride (CCl_4_) is an industrial solvent widely applied in animals to induce liver injury (1). The target organ of CCl_4_ toxicity is not only the liver. Many studies showed that CCl_4_ intoxication in kidney (2-4), blood (5), brain (6), and lung (7) caused free radical generation. Furthermore, Khan *et al.* showed that CCl_4_ causes kidney injury in rats (8). Another study showed that the anti-oxidant status causing nephropathies in rats is altered by the administration of CCl_4_ (9). Furthermore, CCl_4_ caused histopathological features such as inflammation, necrosis, cirrhosis, and tumors (10). The molecular mechanism of CCl_4_ that caused kidney and liver toxicity has been well reported. It is transformed into Cl and trichloromethyl (CCl_3_) radicals through the cytochrome P-450 enzyme system (4). After initiating lipid peroxidation (LPO) in the liver and kidneys, CCl_3_ reacts rapidly with molecular oxygen to produce free radicals. Hydrogen peroxide, superoxide anion, reactive hydroxyl radicals, and peroxyl radicals are the most common Reactive oxygen species (ROS). On the other hand, ROS induces oxidative stress in many diseases, including aging, cancer, and renal diseases (11-13). 

Troxerutin (TRX), a plant flavonoid extracted from the Japanese pagoda tree (*Sophora japonica*) with the chemical formula C_33_H_42_O_19_, is derived from the bioflavonoid rutin and can be found in coffee, tea, and some fruits and vegetables (14, 15). It has anti-oxidant, anti-inflammatory, hepatoprotective, nephroprotective, and antithrombotic effects (16, 17). TRX, also known as vitamin P4, has a protective effect on the liver, brain, kidney, vascular, and heart due to its anti-oxidant and anti-inflammatory effects (18). We examined the protective effect of TRX in the kidney and liver in carbon tetrachloride-intoxicated mice.

## Materials and Methods

### Animal

In this experimental study, 30 male BALB/c mice (30±5 g) were acquired from Rafsanjan University of Medical Sciences Animal House, Rafsanjan, Iran. The male BALB/c mice were kept in a room with 12 hr light/dark cycle, humidity of 45%, and a temperature of 20–23 °C with access to food and water *ad libitum*. The BALB/c mice were maintained in the colony room for one week before starting the experiment. The ethics committee of Rafsanjan University of Medical Sciences approved the study (IR.RUMS.REC.1399.180). Also, this study agreed with the guidelines for animal care and use (National Institutes of Health Publication No. 85-23) revised in 2010. 

### Drugs

CCl_4_ (10 mmol/kg) (Sigma Chemical Co., St. Louis, MO, USA) was freshly dissolved in 50% olive oil (1:1) for intraperitoneal (IP) administration (19). TRX was procured from Sigma-Aldrich Company, Germany. TRX (75 and 150 mg/kg) was prepared daily during the experiment and dissolved in saline solution for oral administration.

### Experimental groups

Mice were randomly divided into five groups (six mice/group) as follows:

Group 1 (Control): This group received olive oil (as a vehicle of CCl_4_) by IP injection on the 1st day, then an hour later, animals were treated with saline (oral gavage) daily for 4 weeks (20).

Group 2 (TRX): This group received olive oil by IP injection on the first day, and then an hour later, animals were treated with TRX (150 mg/kg) by oral gavage daily for 4 weeks (21).

Group 3 (CCl_4_): This group received CCl_4_ (10 mmol/kg) in 50% olive oil (1:1) by IP injection on the first day. An hour later, animals were treated with saline (oral gavage) daily for 4 weeks (19, 20).

Group 4 (CCl_4_ + TRX 75 mg/kg): This group received CCl_4_ (10 mmol/kg) by IP injection on the first day. An hour later, animals were treated with TRX (75 mg/kg) (oral gavage) daily for four weeks.

Group 5 (CCl_4_ + TRX 150 mg/kg): This group received CCl_4_ (10 mmol/kg) by IP injection on the first day. An hour later, animals were treated with TRX (150 mg/kg) (oral gavage) daily for 4 weeks (21).

All groups were given the same volumes of saline or TRX during the experiment. Twenty-four hours after the last administration, mice were anesthetized using ether, and blood samples were taken by heart puncture. Serum samples were prepared via blood centrifugation (6000 g for 15 min). Then, the mice were killed after deep anesthesia, and livers and kidneys were harvested and divided into two parts. One part was kept in formalin (10%) for histological evaluations by Hematoxylin and Eosin (H&E) staining. The other part was homogenized in an ice-cold buffer solution and centrifuged for 20 min at 6000 g. The supernatant was prepared and kept at -80 °C for oxidative parameter evaluation (by the relevant kit) and some biochemical parameters of apoptosis (via the western blotting method). 

### Biochemical parameters

Serum alanine aminotransferase (ALT) and aspartate aminotransferase (AST) levels were measured using the relevant kits (Pars Azmoon Co., Tehran, Iran). Serum creatinine (Cr) and blood urine nitrogen (BUN) levels were determined via quantitative commercial kits (Pars Azmoon Co., Tehran, Iran) (22). 

### Oxidative stress parameters

To evaluate the oxidative stress status in the liver and kidney tissues, the glutathione peroxidase (GPx) and superoxide dismutase (SOD) activity levels, malondialdehyde (MDA) concentration, and total anti-oxidant capacity (TAC) status were measured via available commercial assay kits (ZellBio, Germany) according to the manufacturer’s instructions (23, 24). 

### Apoptosis parameters

The immunoblotting method was performed to evaluate Bax and Bcl-2 protein expression levels as two apoptosis parameters in the liver and kidney tissues. In brief, each protein sample was isolated via 12.5% polyacrylamide gel electrophoresis and subsequently transferred to a polyvinylidene difluoride membrane by electric current. The membranes were incubated overnight (at the temperature of 4 °C and pH 7.4) in Tris-buffered saline and Tween-20 (20 mM Tris–HCl, 150 mM NaCl, 0.1% Tween 20) with 5% nonfat milk. Then, each polyvinylidene difluoride membrane was incubated with rabbit polyclonal anti-Bax, anti-caspase-3, anti-cytochrome-c, and anti-Bcl-2 antibodies (1: 1000, Abcam, USA) for 3 hr at the temperature of 20 to 22 °C. Subsequently, each blot was washed with Tween-20 thrice and incubated with horseradish peroxidase-conjugated anti-rabbit secondary antibody (1:5000, Abcam, USA) at room temperature for another hour. Then, every blot was detected via an enhanced chemiluminescence method. Band densitometry analysis was done using the ImageJ software. Beta (β)-actin immunoblotting (1:5000, Abcam, USA) was applied as a loading control (25).

### Histopathological analysis

The liver and kidney sections were stained using the routine H&E method (magnification 100×). Each stained slide was observed via a light microscope (Nikon Labophot, Japan) in a blind manner to score kidney and liver tissue damage (KTDS and LTDS) for histopathological analysis. We examined six slides per animal to assess tissue damage, focusing on six different areas per slide. Every kidney section was measured for inflammatory cell infiltration, glomerular atrophy, and tubular necrosis. The renal tubulointerstitial injury was evaluated semi-quantitatively according to previous studies (26, 27). Every liver section was measured for congestion and pyknosis. KTDS and LTDS were graded from 0 to 3 by a pathologist blinded to our study (0-0.5= normal, 1= mild, 2= moderate, and 3= severe).

### Data analysis

 Data were analyzed using the GraphPad Prism software package (ver. 6.01, GraphPad Software, USA). The normality of the results was analyzed using the Kolmogorov-Smirnov test. The results are expressed as mean ± SD. For parametric data, one-way ANOVA followed by Tukey’s test was done to evaluate the significance level between the groups. For non-parametric data, the Kruskal-Wallis test was performed. *P*<0.05 is considered a significant level.

## Results

### Effect of troxerutin on biochemical parameters

The biochemical results showed that CCl_4_ increases the levels of the serum Cr (*P*<0.01), BUN (*P*<0.001), AST (*P*<0.001), and ALT (*P*<0.05) indicators in the CCl_4_ group versus the control (Table 1). However, TRX (150 mg/kg) could decrease the serum Cr (*P*<0.05) and BUN (*P*<0.01) levels in the CCl_4_+TRX 150 mg/kg group compared with the CCl_4_ group (Table 1). In addition, TRX (150 mg/kg) significantly decreased the levels of the serum AST and ALT parameters in the TRX 150 mg/kg treated group rather than the CCl_4_ group (*P*<0.05, Table 1).

### Effect of troxerutin on oxidative stress parameters

**Figure 1:**
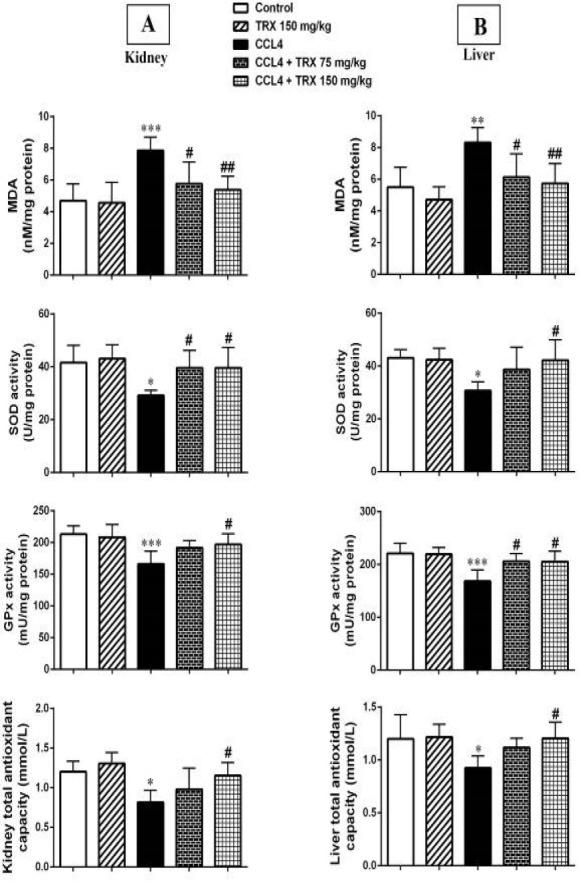
Effect of troxerutin on oxidative stress parameters of kidney (A) and liver (B) tissues in mice with CCl_4_ administration

MDA concentration as an oxidative damage indicator was evaluated after CCl_4_ administration in the kidney and liver tissues of mice. Our result showed that CCl_4_ could significantly increase the MDA concentration in the kidney (*P*<0.001) and liver (*P*<0.01) tissues of CCl_4_-administered mice when compared with the control group. According to results, TRX at doses of 75 and 150 mg/kg significantly decreases the MDA concentration in the kidney (*P*<0.05 and *P*<0.01, respectively) and liver (*P*<0.05 and *P*<0.01, respectively) tissues in the CCl_4_ + TRX 150 mg/kg and CCl_4_ + TRX 75 mg/kg groups compared to the CCl_4_ group (Figure 1). 

The results also showed that CCl_4_ significantly decreases the SOD (*P*<0.05) and GPx (*P*<0.001) activity levels in the kidney and liver tissues of the CCl_4_-administered mice when compared to the control group (Figure 1). TRX at a dose of 150 mg/kg significantly increased the SOD and GPx activity levels in the hepatorenal tissues in the CCl_4_ + TRX 150 mg/kg group compared to the CCl_4_ group (*P*<0.05, Figure 1). In addition, TRX (75 mg/kg) significantly increased the SOD activity level in the kidney tissue of CCl_4_ + TRX 75 mg/kg mice when compared with the CCl_4_ group (*P*<0.05, Figure 1). Also, TRX (75 mg/kg) significantly elevated the GPx activity level in the liver tissue of the CCl_4_ + TRX 75 mg/kg group versus the CCl_4_ group (*P*<0.05, Figure 1).

Our results showed that CCl_4_ significantly decreases the TAC level in kidney and liver tissues in the CCl_4_ group compared to the CCl_4_-administered mice (*P*<0.05, Figure 1). Also, TRX (150 mg/kg) could significantly elevate the TAC level in hepatorenal tissues of the CCl_4_ + TRX 150 mg/kg group rather than the CCl_4_ group (*P*<0.05, Figure 1).

### Effect of troxerutin on hepatorenal tissue apoptosis

**Figure 2:**
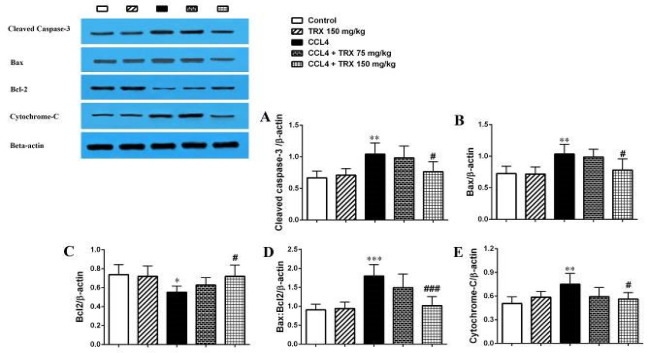
Western blot analysis of the cleaved caspase-3 (A), Bax (B), Bcl-2 (C), Bax/Bcl-2 ratio (D), and cytochrome-c (E) protein expression in the kidney tissue of mice with CCl_4_ administration

**Figure 3:**
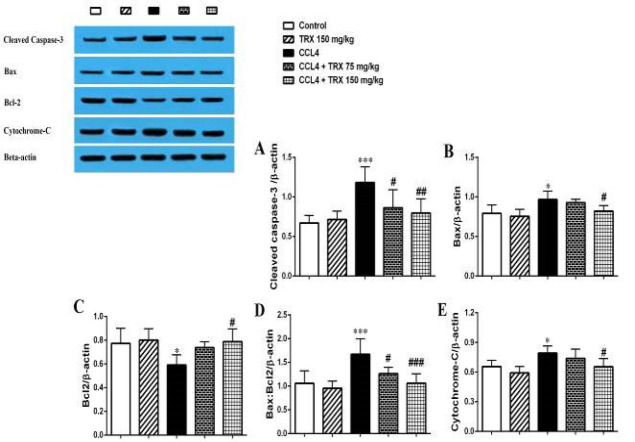
Western blot analysis of the cleaved caspase-3 (A), Bax (B), Bcl-2 (C), Bax/Bcl-2 ratio (D), and cytochrome-c (E) protein expression in the liver tissue of the mice with CCl_4_ administration

Our result revealed that CCl_4_ significantly increases the cleaved caspase-3, Bax, and cytochrome-c proteins’ expression level in the hepatorenal tissues of the CCl_4_ administered mice versus the control group (*P*<0.05, Figures 2 and 3). TRX (150 mg/kg) significantly decreased the cleaved caspase-3, Bax, and cytochrome-c proteins’ expression levels in the hepatorenal tissues of the CCl_4_ + TRX 150 mg/kg group when compared to the CCl_4_ administered mice (*P*<0.05, Figures 2 and 3). In addition, TRX at a dose of 75 mg/kg could significantly decrease the cleaved caspase-3 protein expression level in the liver tissue of the CCL_4_ + TRX 75 mg/kg group versus the CCl_4_ group (*P*<0.05, Figure 3A).

Furthermore, CCl_4_ significantly reduced the Bcl-2 protein expression level in the hepatorenal tissues of the CCl_4_-administered mice versus the control group (*P*<0.05, Figures 2C and 3C). Also, as shown in Figures 2C and 3C, TRX (150 mg/kg) significantly could increase the Bcl-2 protein expression level in the hepatorenal tissues of the CCl_4_ + TRX 150 mg/kg group rather than the CCl_4_ group (*P*<0.05).

Our results also showed that TRX (150 mg/kg) significantly reduced the Bax:Bcl-2 ratio in the hepatorenal tissues of the CCl_4_ + TRX 150 mg/kg group versus the CCl_4 _group. (Figures 2D and 3D,* P*<0.001). However, TRX (150 mg/kg) significantly decreased the Bax:Bcl-2 ratio in the hepatorenal tissues of the CCl_4_ + TRX 150 mg/kg group versus the CCl_4_ group (Figures 2D and 3D,* P*<0.001). In addition, TRX at the dose of 75 mg/kg significantly could decrease the Bax:Bcl-2 ratio in the liver tissue of the CCl_4_ + TRX 75 mg/kg group rather than the CCl_4_ group (Figure 3D,* P*<0.05).

### Effect of troxerutin on hepatorenal tissue damage

**Figure 4:**
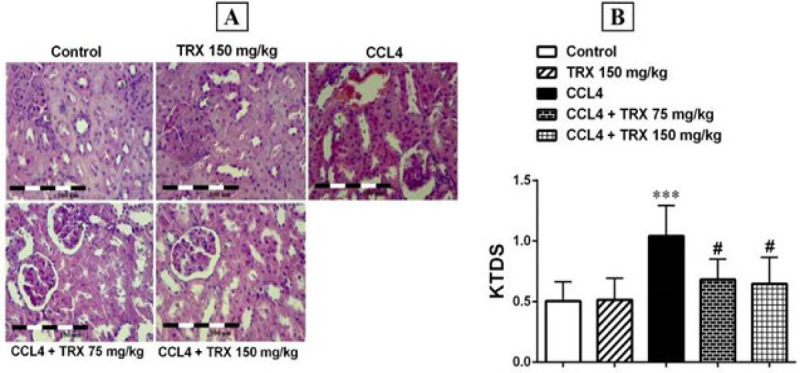
Hematoxylin and Eosin-stained sections (magnification 100×) in the kidney tissue of mice with CCl_4_ administration

**Figure 5:**
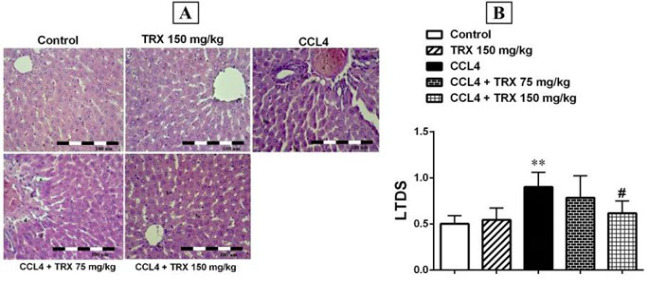
Hematoxylin and Eosin-stained sections (magnification 100×) in the liver tissue of mice with CCl_4_ administration

Based on H&E staining results, no pathologic condition was detected in the hepatorenal tissues of the control mice (Figures 4A and 5A). In addition, the H&E staining results showed that CCl_4_ increases KTDS and LTDS in the CCl_4_ group compared to the control group (*P*<0.01, Figures 4B and 5B), and TRX (150 mg/kg) could ameliorate the damage induced by CCl_4_ in the CCl_4 _+ TRX 150 mg/kg group rather than the CCl_4_ group (*P*<0.05, Figures 4A and 5A). Moreover, KTDS were significantly decreased in the CCl_4_ + TRX 75 mg/kg group versus the CCl_4_ group (*P*<0.05, Figure 4B).

## Discussion

TRX is a semi-synthetic bioflavonoid with different pharmacological properties, including anti-oxidant, anti-inflammatory, and nephroprotective properties (28). Furthermore, it has a protective impact on oxidant damage in the kidney (29) and liver (30). The present study investigated the effect of TRX (75 and 150 mg/kg) on AST, BUN, ALT, and Cr levels and oxidative stress markers against CCl_4_-related hepatorenal damage in mice. 

CCl_4_ treatment increases serum Cr, BUN, ALT, and AST (22). In our study, CCl_4_ nephrotoxicity was confirmed by a significant elevation in serum Cr, BUN, ALT, and AST levels. Furthermore, TRX (150 mg/kg) decreased the CCl_4_-related hepatorenal injuries by a reduction in AST, BUN, Cr, and ALT levels (Table 1). The protective impacts of TRX are partly mediated by its free radical scavenging and anti-oxidant effects. Exposure to CCl_4_ increased the MDA levels produced by the lipid peroxidation (LPO) cascade. Also, our results showed that TRX treatment at 75 and 150 mg/kg decreased oxidative stress via reducing MDA levels, which can be due to the TRX capacity in ROS scavenging (29). On the other hand, our results showed that CCl_4_-treated mice reduced SOD, GPx activities, and TAC in the kidney and liver tissues. Furthermore, TRX at 75 mg/kg increased the GPx, SOD activities, and TRX at 150 mg/kg significantly elevated TAC in the kidney and liver tissues of mice receiving CCl_4_ (Figure 1). This suppression of the activity of anti-oxidant enzymes in the kidney and liver intoxicated with CCl_4_ was inhibited by TRX administration.

In this regard, TRX treatment on the D-galactose-treated mice caused a marked increase in anti-oxidant effects of SOD, GPx, and TAC activities in comparison to D-galactose alone. Thus, the protective effect of TRX can be associated with the high H2O2 scavenging capacity (29).

Shan *et al*. showed that TRX decreased renal urinary protein excretion and cell apoptosis in mice treated with BDE-47. According to western blot analysis, TRX supplementation increased the Bcl-2/Bax ratio. Also, TRX reduced ROS production and increased the anti-oxidative enzyme activities (31). Badalzadeh *et al*. showed that TRX notably lessened the extent of vascular histopathological injuries in diabetic rats that received treatment compared to those that did not. Furthermore, TRX led to a decrease in MDA levels and an increase in GPX activity compared to the untreated diabetic groups. TRX may diminish tissue injuries and vascular complications resulting from chronic diabetes in the rat aorta by enhancing the tissue anti-oxidant system activity and lowering LPO levels (32). Adam *et al*. demonstrated that TRX possesses hepatoprotective qualities, safeguarding the liver against potential coumarin-induced LPO (30).

We found that CCl_4_ can induce apoptosis by an increase in the expression levels of Bax, cleaved caspase-3, and cytochrome-c proteins in the liver and kidney (Figures 2 and 3). Nonetheless, TRX at 150 mg/kg reduced apoptosis in the liver and kidney tissues by lowering the expression levels of Bax, cytochrome-c, and cleaved caspase-3 proteins while increasing the expression level of Bcl-2 protein. Consistent with our findings, Mokhtari *et al*. highlighted the significant role of TRX in suppressing apoptosis factors in liver and kidney tissues (33). Conversely, it is noted that oxidative stress induces apoptosis, leading to degeneration in the liver and kidneys (34). Additionally, our histopathological findings indicated that CCl_4_ exacerbated liver and kidney damage, while TRX (150 mg/kg) was able to alleviate CCl_4_-related liver and kidney tissue damage (Figures 4A and 5A). In our study, pyknosis of hepatocytes, glomerular atrophy, renal tubular necrosis, and renal leukocyte infiltration can confirm hepatorenal damage besides elevated abovementioned biochemical factors in mice receiving CCl_4_. Also, the histological findings of the liver and kidneys align with the aforementioned alterations in the biochemical and oxidative aspects. Consistent with our outcomes, prior research has demonstrated that CCl_4_ triggers oxidative harm and heightens histopathological alterations in liver and kidney tissues (35).

**Table 1:** Effect of troxerutin on kidney and liver functional indicators in mice with CCl_4_ administration

	BUN (mg/dl)	Cr (mg/dl)	AST (U/l)	ALT (U/l)
Group				
Control	48.5±9.77	0.52±0.09	67±9.21	262.2±8.82
TRX 150 mg/kg	43.5±5.54	0.49±0.08	69±7.12	256.8±14.50
CCl_4_	76.5±8.68***	0.73±0.10**	91.8±6.9***	297.5±25.33*
CCl_4 _+ TRX 75 mg/kg	63.2±10.7	0.70±0.09	82±12.7	282.3±20.59
CCl_4_ + TRX 150 mg/kg	53±10.4##	0.56±0.09#	75.3±8.9#	265.2±13.18#

Values are expressed as mean ± SD. n=6 per group. **P˂0.01 versus the control group. #*P*<0.05 and ##*P*<0.01 versus CCl_4_ group

CCl_4_: Carbon tetrachloride, TRX: Troxerutin, BUN: Blood urea nitrogen, Cr: Creatinine, AST: Aspartate aminotransferase, ALT: Alanine transaminase

Values are expressed as mean ± SD. n=6 per group. *P<0.05 and **P<0.01 versus the control group. #*P*<0.05 and ##*P*<0.01 versus the CCl_4_ group. CCl_4_: Carbon tetrachloride, TRX: Troxerutin, MDA: Malondialdehyde, GPx: Glutathione peroxidase, SOD: Superoxide dismutase, TAC: Total antioxidant capacity.

Values are expressed as mean ± SD. n=6 per group. *P<0.05, **P<0.01, and ***P<0.001 versus the control group. #*P*<0.05, ##P<0.01 and ###*P*<0.001 versus CCl_4_ group. CCl_4_: Carbon tetrachloride, TRX: Troxerutin.

Values are expressed as mean ± SD. n=6 per group. *P<0.05, **P<0.01, and ***P<0.001 versus the control group. #*P*<0.05, ##*P*<0.01 and ###*P*<0.001 versus CCl_4_ group. CCl_4: _Carbon tetrachloride, TRX: Troxerutin.

A: 'a' signifies normal glomeruli, 'b' signifies normal tubules, 'c' signifies leucocyte infiltration, 'd' signifies glomerular atrophy, and 'e' signifies tubular atrophy. B: Kidney tissue damage score (KTDS) in CCl_4_-induced hepatorenal damage mice four weeks after administration of saline/TRX. Scores 1–3 were considered as leukocyte infiltration, glomerular atrophy, and tubular necrosis in the kidney tissue sections. However, scores 0 to 0.5 were considered normal tissue. Values are expressed as mean ± SD. n=6 per group. Arrow: glomerular atrophy/tubular necrosis; Circle: leukocyte infiltration.***P<0.001 versus the control group. #*P*<0.05 versus CCl_4_ group. CCl_4_: Carbon tetrachloride, TRX: Troxerutin.

A: ‘a’ indicates dilated sinusoids, ‘b’ indicates pyknosis, ‘c’ indicates congestion, and 'd' indicates fat deposit. B: Scores 1–3 were considered congestion and pyknotic cells in the liver tissue sections. However, scores 0 to 0.5 were considered normal tissue. Liver tissue damage score (LTDS) in CCl_4_-induced hepatorenal damage mice four weeks after administration of saline/TRX. Values are expressed as mean ± SD. n=6 per group. ***P<0.001 versus the control group. Arrow: pyknosis/fat deposit; Circle: congestion. ##*P*<0.01 versus CCl_4_ group. CCl_4_: Carbon tetrachloride TRX: Troxerutin.

## Conclusion

The current research indicates that the flavonoid TRX exhibits potential in protecting the liver and kidneys. It efficiently combats oxidative damage induced by CCl_4_, mitigating tissue damage and apoptosis in the liver and kidneys by preserving the endogenous anti-oxidant system and neutralizing free radicals.

## Data Availability

Data could be obtained upon request from the corresponding author.

## Declaration

We have not used any AI tools or technologies to prepare this manuscript.
